# Supplementary material for: Multispecies Bacterial Bio-Input: Tracking and Plant-Growth-Promoting Effect on Lettuce var. sagess
Source: Plants (Basel). 2023 Feb 7;12(4):736. doi: 10.3390/plants12040736 (PMC9962684; doi:10.3390/plants12040736)
Supplement: Supplementary file 1 [file plants-12-00736-s001.zip › plants-2158230-supplementary.pdf]

Supplementary Materials

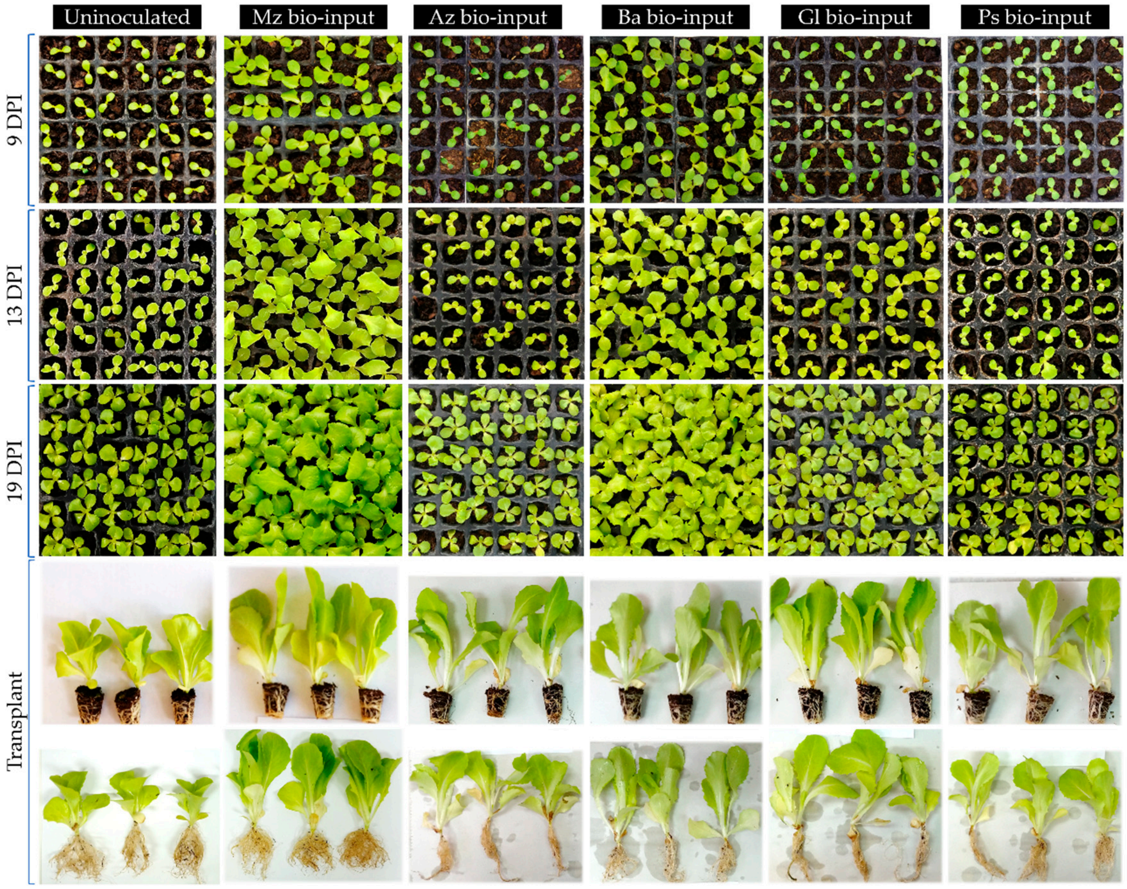

Figure S1. Photographs of lettuce seedlings grown in controlled conditions several days post-inoculation (DPI).
